# Supplementary material for: Clonal haematopoiesis harbouring AML-associated mutations is ubiquitous in healthy adults
Source: Nat Commun. 2016 Aug 22;7:12484. doi: 10.1038/ncomms12484 (PMC4996934; doi:10.1038/ncomms12484)
Supplement: Supplementary Information — Supplementary Figures 1-10 and Supplementary Tables 1-6. [file ncomms12484-s1.pdf]

Supplementary Figure 1 Coverage per amplicon for error-corrected sequencing experiments. Error-corrected consensus sequence (ECCS) coverage was calculated for each of the 568 amplicons in the capture panel. **a,** Histogram of ECCS coverage for all amplicons. **b,** Histogram of ECCS coverage in amplicons in which a variant was detected.

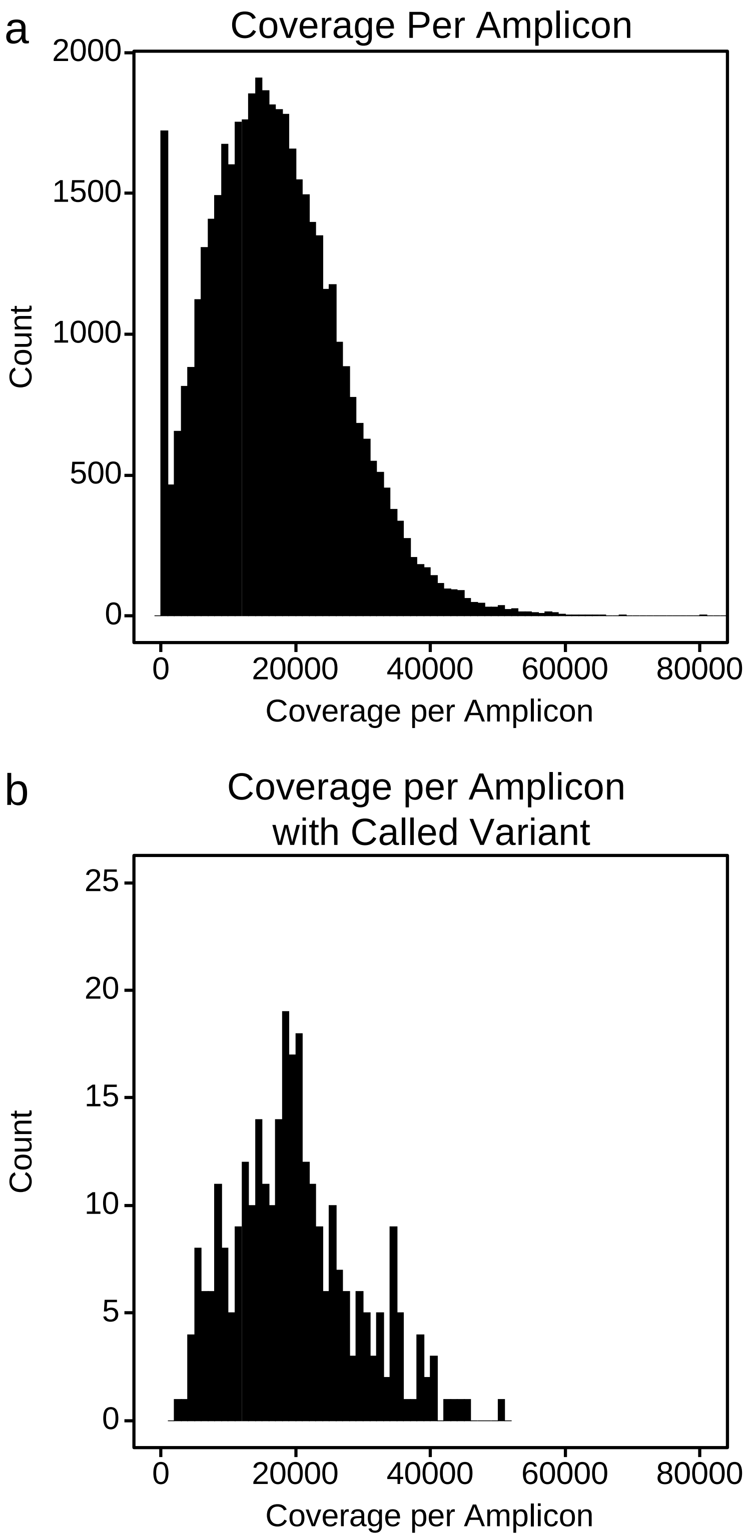

Supplementary Figure 2 Number of mutations detected per gene in exons (top panel) and introns (bottom panel) relative to the capture space (bp = base pairs) targeting that gene in the panel.

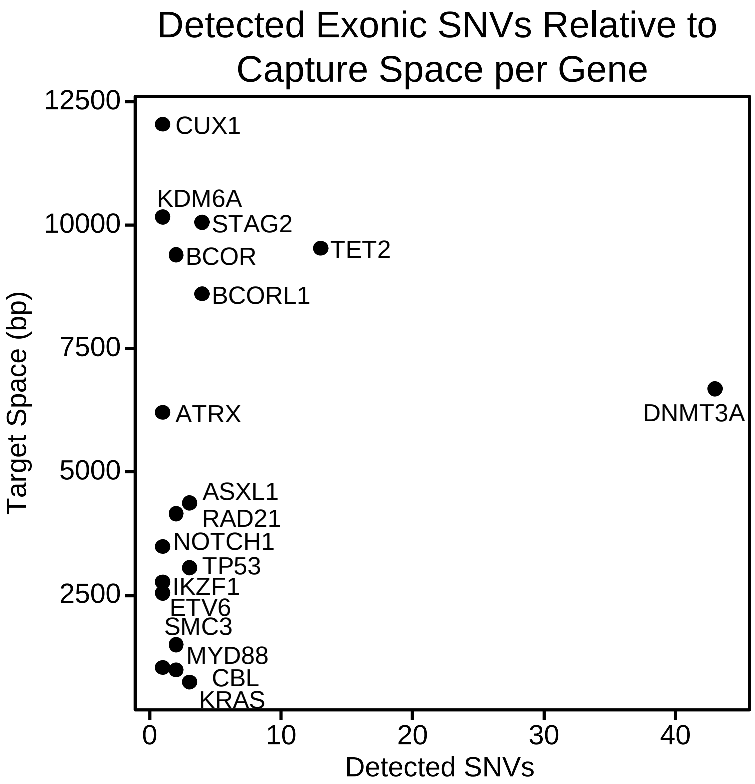

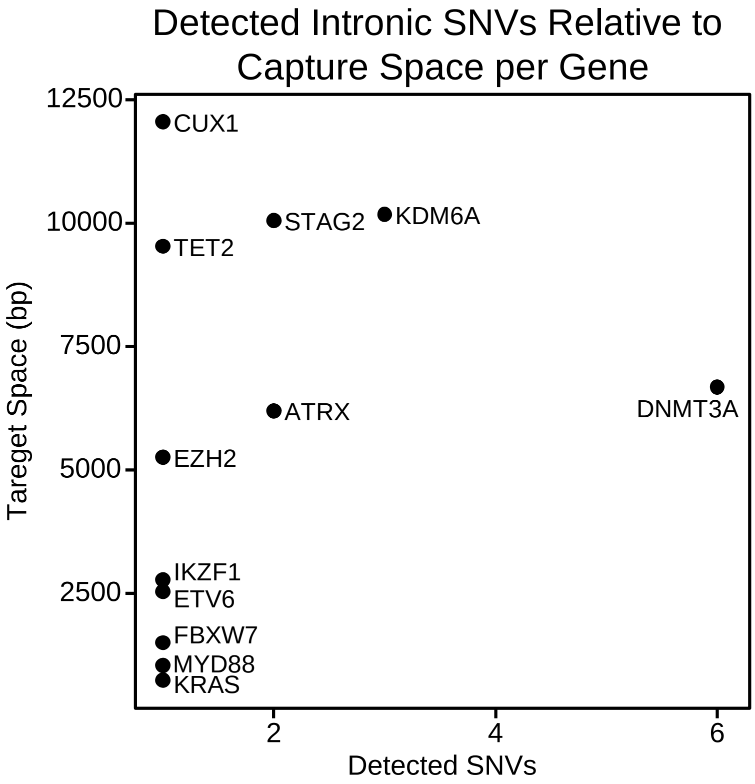

Supplementary Figure 3 Representative droplet digital PCR (ddPCR) results from NHS participant 12 for the detected *DNMT3A* G543A clonal variant. The wild-type probe intensity in arbitrary units (A.U.) was plotted relative to the *DNMT3A* G543A (mutant) probe intensity for each droplet. **a-d,** Variant quantification at the first time point for **a,** all cells; **b,** B lymphocytes; **c,** T lymphocytes; and **d,** myeloid cells. **e-h,** Variant quantification at the second time point for **e,** all cells; **f,** B lymphocytes; **g,** T lymphocytes; and **h,** myeloid cells. **i,** The *DNMT3A* G543A variant was not detected in the negative control sample from participant 2, time point 2. **j,** Only DNMT3A G543A positive (or empty) droplets were detected in the gblock positive control.

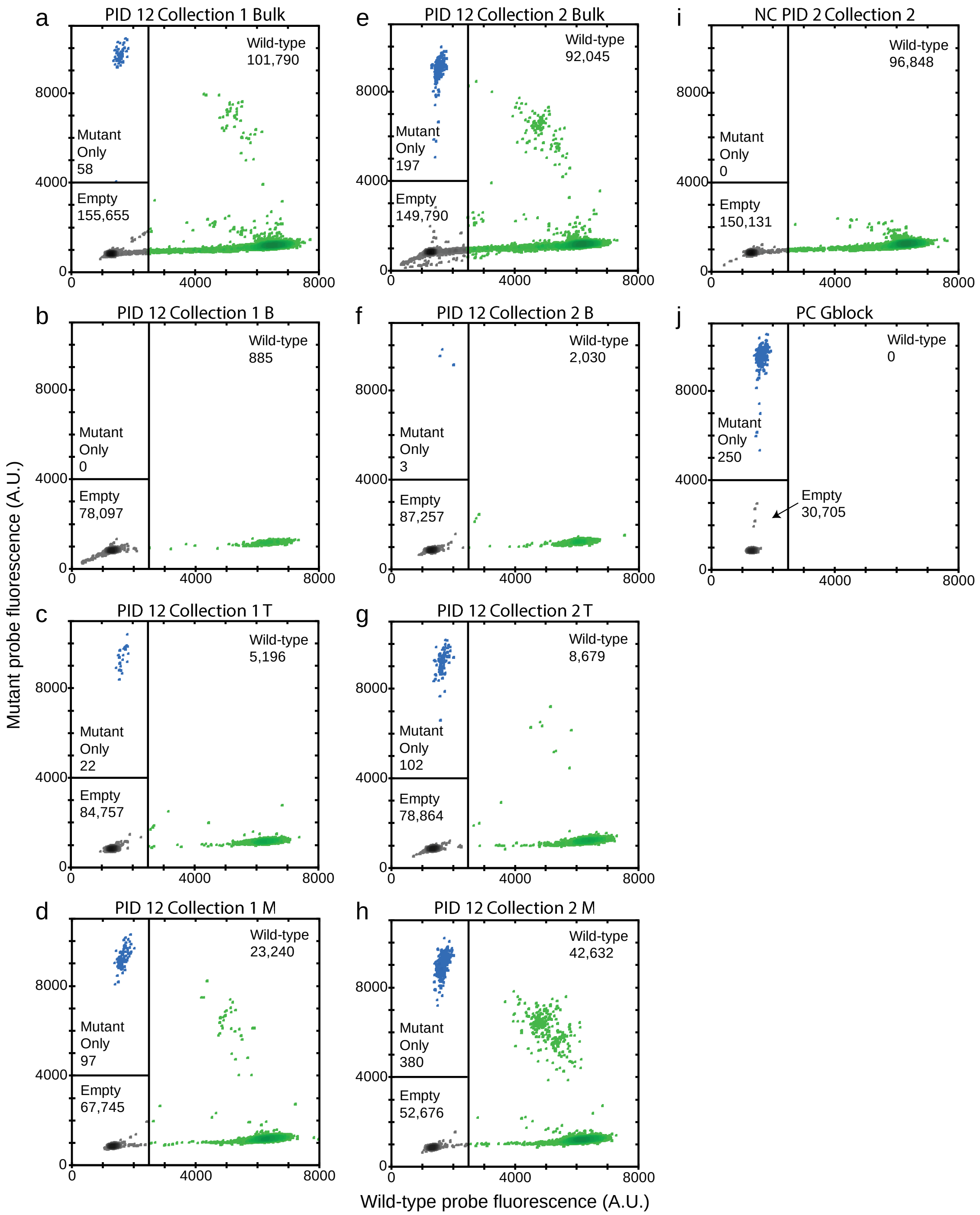

Supplementary Figure 4. Several mutations identified by error-corrected sequencing (ECS) were verified using droplet digital PCR (ddPCR). The variant allele fractions (VAFs) identified ECS and ddPCR were highly correlated (R^2=0.98).

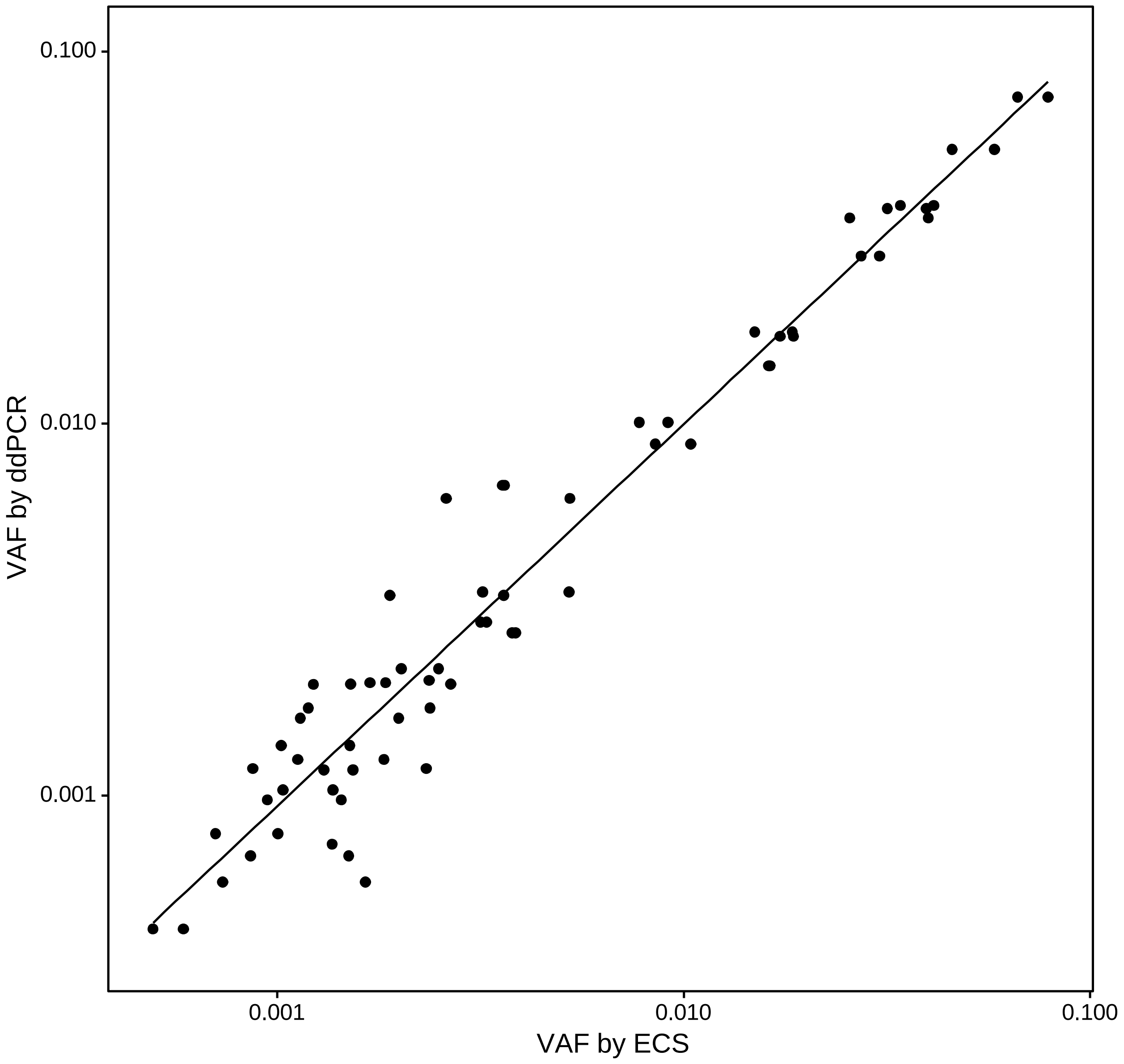

Supplementary Figure 5 Heat map depicting the number of exonic single nucleotide variants (SNVs) detected in each gene per study participant.

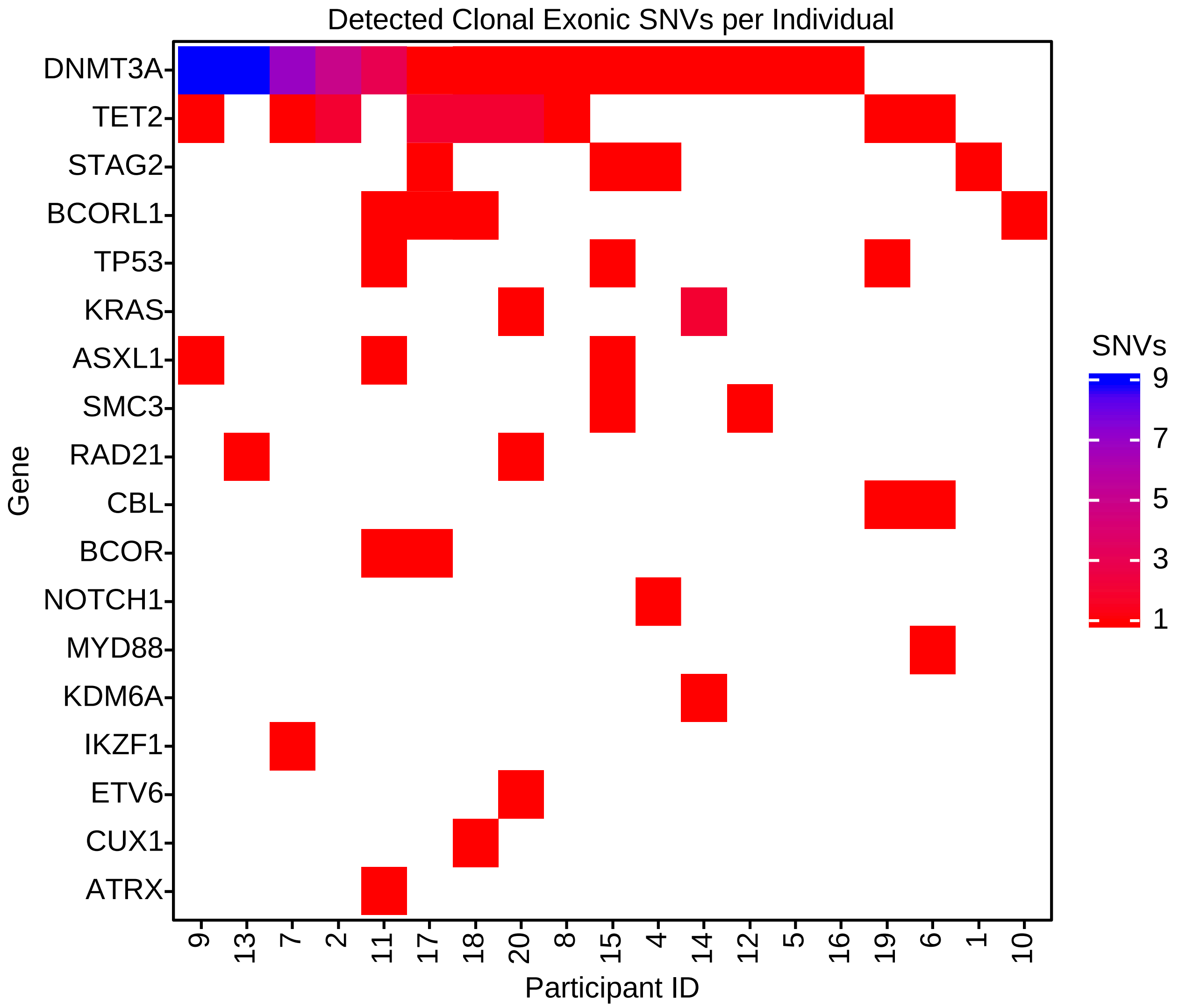

Supplementary Figure 6 Detected exonic clonal single nucleotide variants (SNVs) in *DNMT3A*. The detected SNVs were predominantly nonsense mutations (blue) in the first half of the gene or missense mutations (red) in the three functional domains—a proline-tryptophan-tryptophan-proline (PWWP) chromatin targeting domain, a zinc finger nuclease (ZFN) domain and a S-adenosylmethionine (SAM) dependent methyltransferase (MTase) domain.

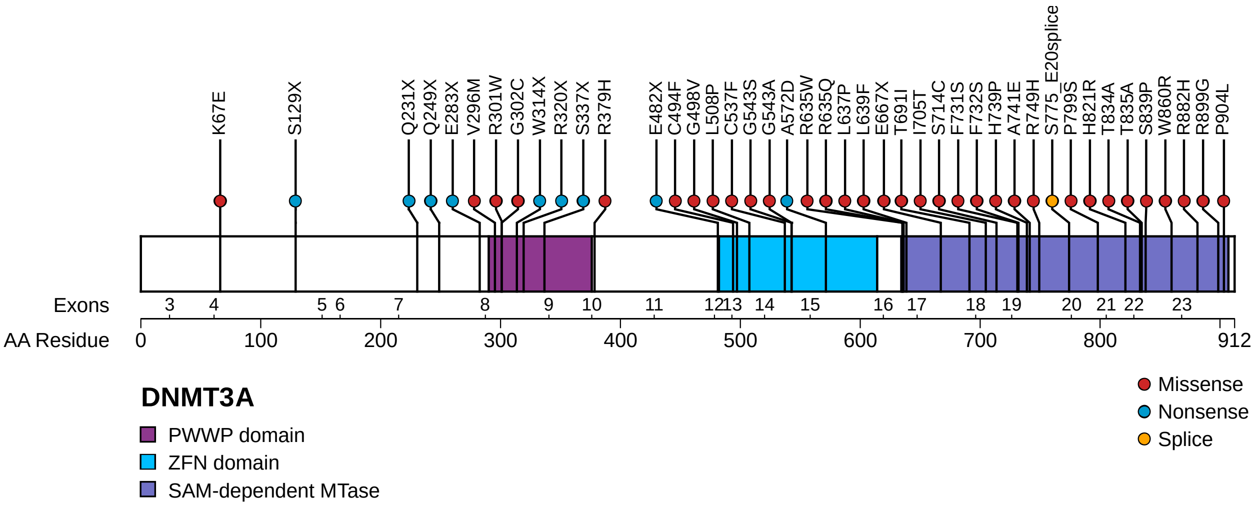

Supplementary Figure 7 Detected exonic clonal single nucleotide variants in *TET2.*

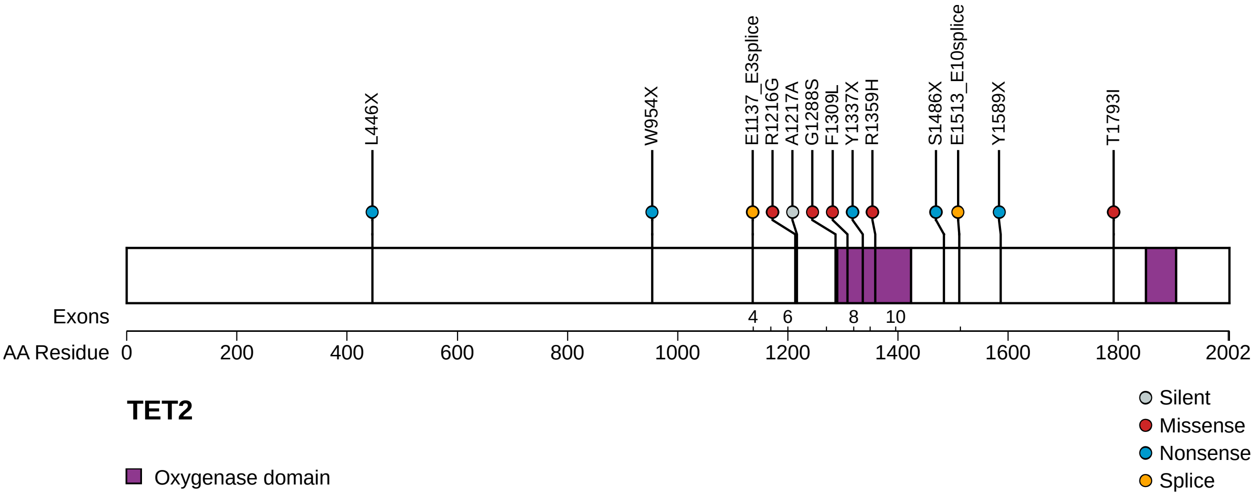

Supplementary Figure 8 Number of intronic clonal single nucleotide variants (SNVs) detected by gene.

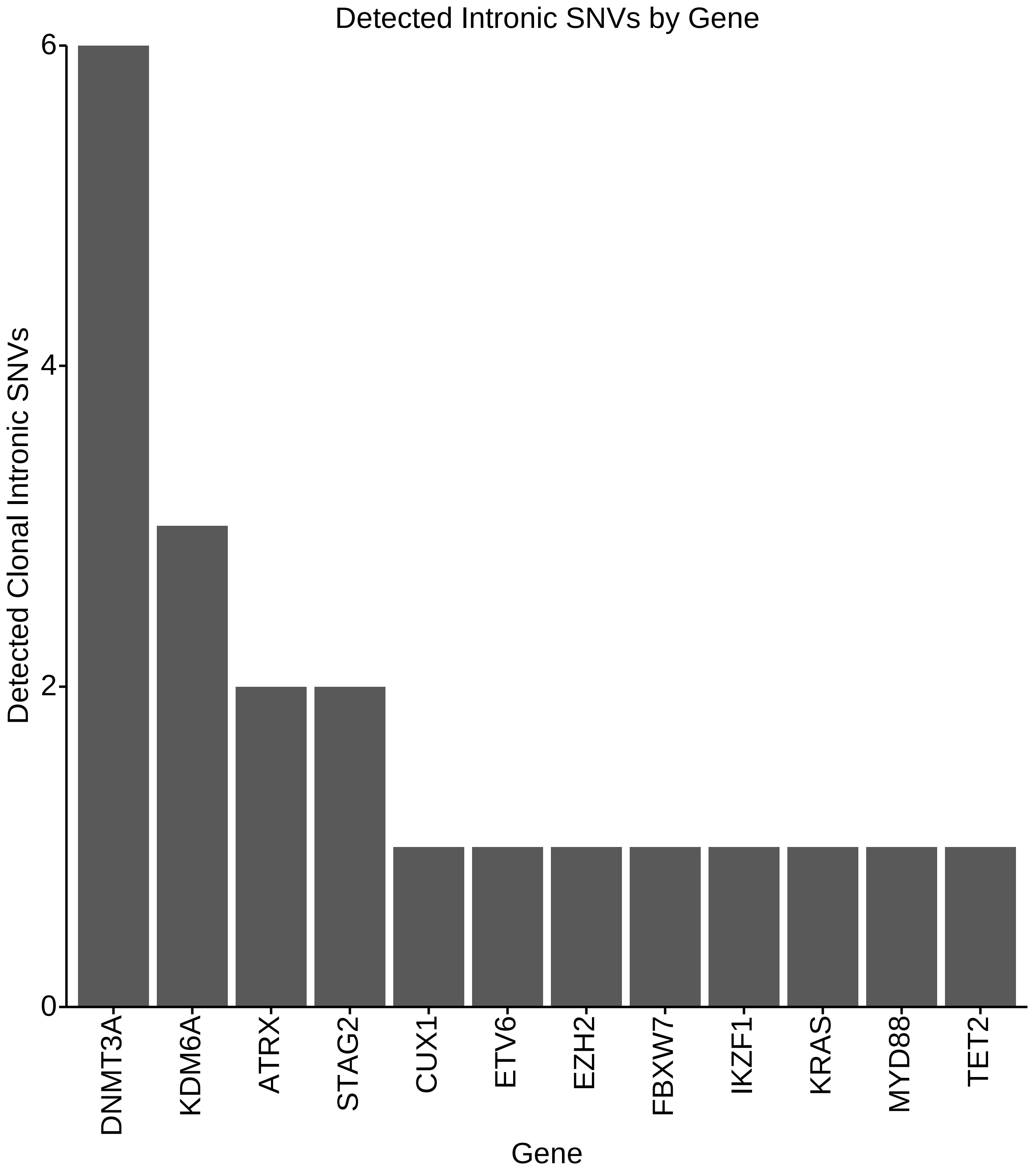

Supplementary Figure 9 Heat map depicting the number of intronic single nucleotide variants (SNVs) detected in each gene per individual.

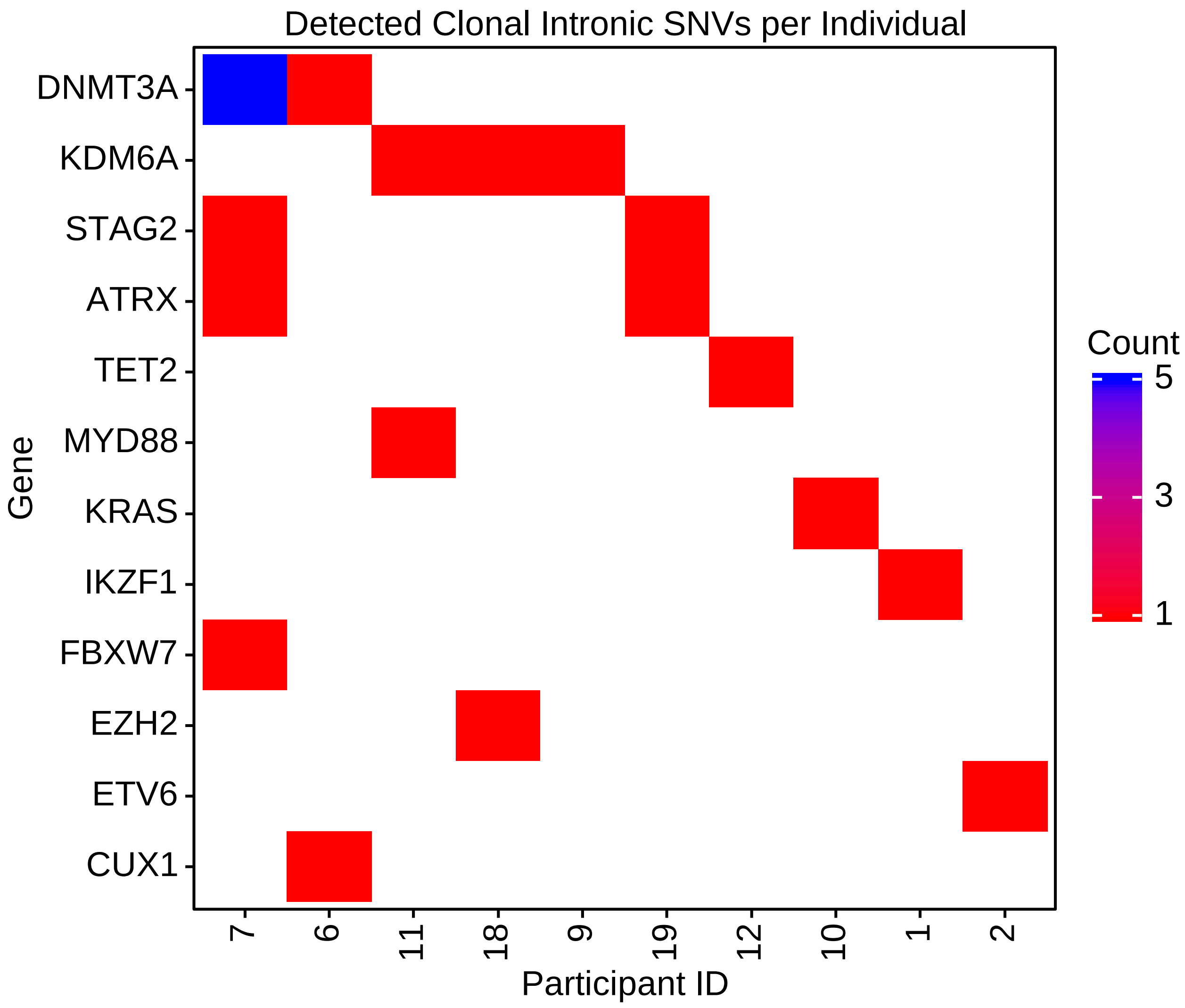

Supplementary Figure 10 Representative flow cytometry gating strategy from NHS participant 5, time point 1 to isolate B lymphocytes, T lymphocytes and myeloid cells.

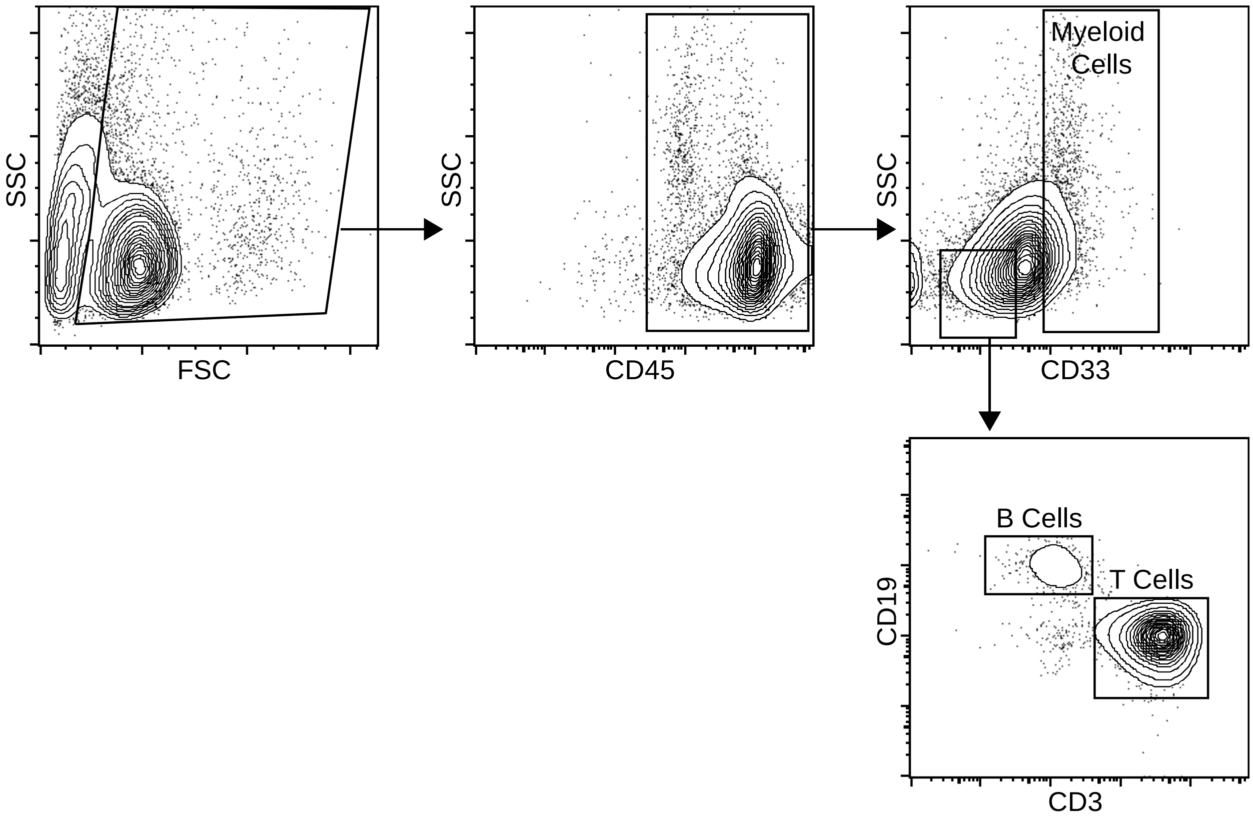

Supplementary Table 1 Sequenced reads and error-corrected consensus sequences generated for each library.

	Collection 1 Replicate 1		Collection 1 Replicate 2		Collection 2 Replicate 1		Collection 2 Replicate 2	
**Participant ID**	**Raw Reads**	**ECCS**	**Raw Reads**	**ECCS**	**Raw Reads**	**ECCS**	**Raw Reads**	**ECCS**
1	38,483,255	3,333,626	33,448,218	2,804,567	35,427,539	2,987,274	33,392,978	2,784,229
2	39,268,072	3,318,173	41,984,812	3,558,516	35,157,811	3,027,607	33,657,209	2,860,180
3	32,603,819	2,581,039	36,107,671	2,959,152	48,998,142	3,584,046	40,599,238	3,291,215
4	30,932,163	2,212,764	30,623,846	2,501,632	39,579,433	3,254,544	48,529,452	3,503,765
5	35,011,143	2,727,030	34,151,207	2,411,821	52,302,285	3,759,106	55,049,072	4,017,037
6	34,207,169	2,863,690	35,084,657	2,946,669	50,852,817	3,682,303	48,351,486	3,514,019
7	41,658,678	2,663,917	42,508,068	2,714,869	45,885,262	3,233,300	44,468,353	3,548,708
8	44,771,597	2,734,288	41,632,517	2,528,357	50,072,031	3,399,553	50,378,471	3,698,270
9	39,449,116	2,531,229	41,067,140	2,599,127	60,014,462	4,197,532	50,347,145	3,993,077
10	40,492,765	2,554,060	38,729,489	2,400,500	59,870,612	4,034,423	58,962,293	3,996,550
11	48,940,303	3,684,038	44,034,692	3,456,949	64,520,183	4,096,893	56,501,287	3,797,404
12	57,115,177	4,245,185	48,446,875	3,692,857	61,813,583	4,322,748	59,452,070	4,110,288
13	39,368,839	3,059,660	41,269,631	3,343,408	59,327,495	4,213,628	52,689,305	4,173,008
14	38,837,743	3,076,601	37,306,017	2,976,419	60,366,370	4,053,326	58,109,532	4,501,213
15	54,605,075	3,407,283	44,547,457	2,490,226	58,101,101	3,908,542	51,539,869	4,104,014
16	52,226,742	2,986,829	60,744,391	3,907,372	45,532,881	3,414,608	60,632,143	4,027,633
17	57,985,852	4,079,429	51,835,232	3,373,746	55,355,241	3,721,052	59,501,527	4,096,858
18	54,185,217	3,337,380	54,495,083	3,388,504	57,117,288	4,147,180	58,064,485	3,999,074
19	52,213,946	2,947,031	51,028,264	3,343,682	53,983,868	3,723,161	48,268,843	3,796,565
20	51,521,626	3,376,026	41,417,619	2,806,990	58,651,106	4,092,482	56,692,728	3,793,691

Supplementary Table 2 Clonal SNVs detected by error-corrected sequencing.

Participant ID	Chr	Start	End	Ref	Alt	Gene	Amino Acid	COSMIC	VAF1.1	VAF1.2	VAF2.1	VAF2.2
1	7	50444517	50444517	T	C	IKZF1	intronic		0.000922	-	0.001021	0.002929
1	X	123185174	123185174	G	T	STAG2	V376L		0.002824	0.001194	0.018021	0.016985
2	2	25463271	25463271	G	T	DNMT3A	A741E		0.001124	-	0.000988	0.001011
2	2	25463277	25463277	T	G	DNMT3A	H739P		0.001713	0.000284	0.000365	0.000533
2	2	25466793	25466793	A	G	DNMT3A	L637P		0.00136	0.003425	0.003549	0.005639
2	2	25468153	25468153	A	G	DNMT3A	L508P		-	0.001168	0.007558	0.008506
2	2	25470573	25470573	G	A	DNMT3A	R301W		-	-	0.002601	0.003542
2	4	106164778	106164778	C	G	TET2	R1216G		-	-	0.00106	0.001038
2	4	106164783	106164783	T	A	TET2	A1217A		-	-	0.001053	0.001032
2	12	11803160	11803160	G	C	ETV6	intronic		0.001224	0.000567	-	-
4	2	25470516	25470516	G	A	DNMT3A	R320X	133724	0.00363	0.00358	0.005249	0.002602
4	9	139391179	139391179	G	A	NOTCH1	Q2338X		0.001138	0.000967	-	-
4	X	123184056	123184056	G	T	STAG2	R305L	254953	0.009143	0.007774	0.014936	0.018524
5	2	25466799	25466799	C	T	DNMT3A	R635Q	1583088	-	-	0.001517	0.002674
6	2	25471183	25471183	C	G	DNMT3A	intronic		0.001627	0.001534	-	-
6	3	38181952	38181952	C	T	MYD88	I192I		-	-	0.00177	0.001559
6	4	106180899	106180899	T	G	TET2	F1309L		0.001359	0.001286	0.000901	0.000766
6	7	101843537	101843537	T	G	CUX1	intronic		0.004012	0.004168	0.001704	0.005179
6	11	119148929	119148929	A	G	CBL	I383M		0.001394	0.000438	-	-
7	2	25457176	25457176	G	A	DNMT3A	P904L	87007	0.002364	-	0.008514	0.010401
7	2	25458673	25458673	T	C	DNMT3A	T834A		0.002895	0.003496	0.002633	0.003961
7	2	25462012	25462012	G	A	DNMT3A	P799S		-	-	0.002258	0.004782
7	2	25463372	25463372	G	A	DNMT3A	intronic		0.004215	0.003861	0.003416	-
7	2	25463384	25463384	G	A	DNMT3A	intronic		0.006557	0.0082	0.002896	-
7	2	25463385	25463385	C	G	DNMT3A	intronic		-	-	0.006618	0.008666
7	2	25463387	25463387	C	G	DNMT3A	intronic		0.006654	0.007867	0.003217	-
7	2	25463389	25463389	G	A	DNMT3A	intronic		0.006822	0.007738	0.003792	-
7	2	25464441	25464441	G	A	DNMT3A	T691I		-	-	0.00358	0.002517
7	2	25464514	25464514	C	A	DNMT3A	E667X		-	-	0.001078	0.003162
7	2	25466788	25466788	G	A	DNMT3A	L639F		0.021615	0.020599	0.040688	0.029481
7	2	25467449	25467449	C	T	DNMT3A	G543S		-	-	0.004758	0.003253
7	4	106158509	106158509	G	A	TET2	splicing	87117	-	-	0.001024	0.001509
7	4	153249632	153249632	T	C	FBXW7	intronic		0.00035	0.000329	-	-
7	7	50367256	50367256	C	T	IKZF1	S21S		0.003464	0.004061	0.002104	-
7	X	76874262	76874262	A	T	ATRX	intronic		-	-	0.00046	0.000325
7	X	123199914	123199914	G	T	STAG2	intronic		-	-	0.000751	0.000892
8	2	25468919	25468919	C	A	DNMT3A	E482X		-	-	0.00305	0.004007
8	4	106180834	106180834	G	A	TET2	G1288S	110780	0.001193	0.002377	0.001991	0.00114
9	2	25459821	25459821	T	C	DNMT3A	H821R		-	0.002221	0.001677	0.002502
9	2	25462086	25462086	T	C	DNMT3A	splicing		0.002556	-	0.001467	0.001506
9	2	25463247	25463247	C	T	DNMT3A	R749H		0.020272	0.017677	0.033274	0.035612
9	2	25463541	25463541	G	C	DNMT3A	S714C	87011	0.027327	0.030296	0.04122	0.034103
9	2	25466800	25466800	G	A	DNMT3A	R635W	87012	0.001896	0.003609	-	0.001227
9	2	25467160	25467160	G	T	DNMT3A	A572D		-	-	0.001003	0.002101
9	2	25469632	25469632	C	T	DNMT3A	R379H		-	-	0.002941	0.005707
9	2	25470588	25470588	C	T	DNMT3A	V296M		-	-	0.003471	0.003189
9	2	25471016	25471016	G	A	DNMT3A	Q249X		0.00142	-	0.001329	0.001299
9	4	106193995	106193995	C	G	TET2	S1486X	211625	-	-	0.000495	0.000588
9	20	31023091	31023091	A	G	ASXL1	N859S		0.004057	0.002914	0.00438	0.003188
9	X	44733267	44733267	T	G	KDM6A	intronic		-	-	0.001671	0.001338
10	12	25380459	25380459	G	C	KRAS	intronic		-	-	0.001002	0.00086
10	X	129162659	129162659	C	G	BCORL1	H1376Q		0.004852	0.002344	0.00182	-
11	2	25458595	25458595	A	G	DNMT3A	W860R	231568	0.000861	0.0015	0.002019	0.002493
11	2	25470914	25470914	C	A	DNMT3A	E283X		0.004158	0.005587	0.012941	0.014349
11	2	25505372	25505372	G	C	DNMT3A	S129X		-	-	0.000727	0.000548
11	3	38182245	38182245	T	A	MYD88	intronic		-	-	0.001872	0.002152
11	17	7577129	7577129	A	G	TP53	F270S	11305	-	-	0.000734	0.001649
11	20	31023606	31023606	A	T	ASXL1	K1031X		0.000673	0.000843	-	-
11	X	39933358	39933358	G	A	BCOR	A414V		-	-	0.000583	0.000915
11	X	44733249	44733249	G	T	KDM6A	intronic		-	-	0.000883	0.000815
11	X	76814150	76814150	A	T	ATRX	F2165Y		0.000715	0.000746	-	-
11	X	129148158	129148158	A	G	BCORL1	L470L		-	-	0.000251	0.000509
12	2	25467448	25467448	C	G	DNMT3A	G543A	256033	0.001365	-	0.003789	0.003858
12	4	106155048	106155048	C	G	TET2	intronic		-	-	0.000993	0.001965
12	10	112342324	112342324	C	T	SMC3	S243F		-	-	0.001413	0.001779
13	2	25457192	25457192	G	C	DNMT3A	R899G		0.002495	0.0015	0.001342	0.001758
13	2	25457242	25457242	C	T	DNMT3A	R882H	52944	-	-	0.001847	0.001692
13	2	25458658	25458658	A	G	DNMT3A	S839P		0.000346	-	0.001094	0.000638
13	2	25463298	25463298	A	G	DNMT3A	F732S		0.001517	-	0.002979	0.003191
13	2	25467466	25467466	C	A	DNMT3A	C537F		-	-	0.001861	0.003427
13	2	25468183	25468183	C	A	DNMT3A	G498V		0.000907	-	0.003704	0.006033
13	2	25468195	25468195	C	A	DNMT3A	C494F		0.000907	-	0.003706	0.006041
13	2	25470532	25470532	C	T	DNMT3A	W314X		-	-	0.002078	0.002564
13	2	25470570	25470570	C	A	DNMT3A	G302C		-	-	0.001633	0.000994
13	8	117859842	117859842	T	C	RAD21	Y598C		-	-	0.000507	0.000562
14	2	25470464	25470464	G	C	DNMT3A	S337X		0.025249	0.023997	0.042269	0.044835
14	12	25380260	25380260	T	G	KRAS	A66A		-	-	0.00628	0.005504
14	12	25380263	25380263	A	G	KRAS	S65S		-	-	0.006442	0.005566
14	X	44911015	44911015	C	T	KDM6A	A239V		-	-	0.004325	0.005592
15	2	25458670	25458670	T	C	DNMT3A	T835A		-	-	0.000534	0.000511
15	10	112342321	112342321	T	C	SMC3	L242P		0.002797	0.00341	0.003635	0.004656
15	17	7577105	7577105	G	T	TP53	P278H	43755	0.016175	0.016297	0.031688	0.039548
15	20	31024085	31024085	G	T	ASXL1	R1190S		-	-	0.001109	0.000538
15	X	123234540	123234540	T	A	STAG2	UTR3		-	-	0.000661	0.000639
16	2	25463568	25463568	A	G	DNMT3A	I705T	1583102	0.066263	0.078765	0.045716	0.058166
17	2	25463301	25463301	A	G	DNMT3A	F731S		0.001212	-	0.001883	0.002564
17	4	106156436	106156436	T	G	TET2	L446X		-	-	0.000747	0.000934
17	4	106197045	106197045	C	T	TET2	T1793I		-	-	0.001308	0.001864
17	X	39932643	39932643	G	A	BCOR	P652P		0.005509	0.005769	0.001855	0.002876
17	X	123224536	123224536	A	T	STAG2	K1130I		0.000814	0.000899	0.000919	0.00098
17	X	129173203	129173203	G	A	BCORL1	G1522S		-	-	0.000448	0.000444
18	2	25471070	25471070	G	A	DNMT3A	Q231X		0.001527	0.001449	0.001864	0.000894
18	4	106157961	106157961	G	A	TET2	W954X	87110	-	-	0.002327	0.000871
18	4	106182972	106182972	T	A	TET2	Y1337X	87145	-	-	0.001537	0.001304
18	7	101840496	101840496	G	A	CUX1	R602H		0.147878	0.142414	0.082825	0.06929
18	7	148515272	148515272	A	G	EZH2	intronic		-	-	0.001265	0.001335
18	X	44938634	44938634	A	G	KDM6A	intronic		0.013796	0.012084	0.014613	0.013679
18	X	129149098	129149098	C	T	BCORL1	R784X	1319521	-	-	0.018627	0.017259
19	4	106196434	106196434	T	G	TET2	Y1589X		-	0.002147	0.002257	0.002473
19	11	119148922	119148922	G	A	CBL	C381Y	34073	0.001034	0.001371	0.001124	0.001829
19	17	7578427	7578427	T	C	TP53	H168R	43545	0.000705	0.001006	0.003275	0.003164
19	X	76813170	76813170	A	G	ATRX	intronic		-	-	0.000688	0.001001
19	X	123179344	123179344	C	T	STAG2	intronic		0.01895	0.015979	0.020188	0.02219
20	2	25505559	25505559	T	C	DNMT3A	K67E		0.005155	0.008004	0.007605	0.010001
20	4	106190798	106190798	G	A	TET2	R1359H		0.003494	0.003217	-	-
20	4	106194076	106194076	G	A	TET2	splicing		-	-	0.00145	0.001679
20	8	117878873	117878873	C	A	RAD21	V32V		0.004314	0.003977	0.00754	0.007097
20	12	12022854	12022854	C	A	ETV6	V320V		-	-	0.001038	0.001637
20	12	25398284	25398284	C	T	KRAS	G12D	521	-	-	0.000946	0.001437

Supplementary Table 3 Clonal insertion/deletion variants detected by error-corrected sequencing.

Participant ID	Chr	Start	End	Ref	Alt	Gene	AA_Change	VAF1.1	VAF1.2	VAF2.1	VAF2.2
7	2	25463381	25463381	-	GTG	DNMT3A	intronic	0.006769	0.007010	0.003021	-
7	4	106155858	106155861	CAGT	-	TET2	N253fs	0.006301	0.006283	0.013260	0.012641
7	4	106164895	106164895	-	A	TET2	Y1255_G1256delinsX	0.007199	0.005162	0.007387	0.008972
9	2	25463567	25463567	A	-	DNMT3A	I705fs	0.033141	0.022850	0.060062	0.080059
9	2	25467528	25467547	AGCAGCGGGAAGGGTCAGAA	-	DNMT3A	intronic	-	-	0.003843	0.004093
11	2	25468168	25468168	-	T	DNMT3A	T503fs	-	-	0.002980	0.003126
15	8	117862892	117862895	TCTC	-	RAD21	E528fs	0.006811	0.006486	0.014212	0.012793
18	X	123179310	123179318	ATTAATTTT	-	STAG2	intronic	0.027702	0.025452	0.119350	0.120730
20	4	106190864	106190864	C	-	TET2	A1381fs	0.005228	0.003205	0.039980	0.025601

Supplementary Table 4 Summary of droplet digital PCR validation experiments.

					Collection 1			Collection 2				
Participant ID	Gene	AA_Change	Cosmic	Wells	ECS VAF Replicate 1	ECS VAF Replicate 2	ddPCR VAF	ECS VAF Replicate 1	ECS VAF Replicate 2	ddPCR VAF	ddPCR Control UPN.Collection	Control VAF
4	DNMT3A	R320X	133724	16	0.003630	0.003580	0.006820	0.005249	0.002602	0.006283	1016078.2	0.000000
4	STAG2	R305L	254953	8	0.009143	0.007774	0.010077	0.014936	0.018524	0.017639	1016078.1	0.000000
5	DNMT3A	R635Q	1583088	32	-	-	0.000938	0.001517	0.002674	0.001993	1819476.1	0.000006
7	DNMT3A	P904L	87007	16	0.002364	-	0.002043	0.008514	0.010401	0.008802	1038614.1	0.000025
7	TET2	splice	87117	24	-	-	0.000856	0.001024	0.001509	0.001363	1038614.2	0.000000
8	TET2	G1288S	110780	16	0.001193	0.002377	0.001720	0.001991	0.001140	0.001615	101785A.1	0.000014
9	DNMT3A	S714C	87011	8	0.027327	0.030296	0.028169	0.041220	0.034103	0.038544	101785A.2	0.000024
9	DNMT3A	R635W	87012	16	0.001896	0.003609	0.003453	-	0.001227	0.001991	1081785.2	0.000000
9	TET2	S1486X	211625	24	-	-	-	0.000495	0.000588	0.000438	1060647.1	0.000000
11	TP53	F270S	11305	24	-	-	0.000457	0.000734	0.001649	0.000586	101785A.1	0.000000
11	DNMT3A	W860R	231568	24	0.000861	0.001500	0.000689	0.002019	0.002493	0.002193	1060647.2	0.000000
12	DNMT3A	G543A	256033	16	0.001365	-	0.000740	0.003789	0.003858	0.002741	101785A.2	0.000000
13	DNMT3A	R882H	52944	24	-	-	0.000216	0.001847	0.001692	0.002011	1115442.1	0.000009
15	TP53	P278H	43755	8	0.016175	0.016297	0.014296	0.031688	0.039548	0.037802	1081785.2	0.000074
16	DNMT3A	I705T	1583102	8	0.066263	0.078765	0.075309	0.045716	0.058166	0.054531	1115442.1	0.000000
18	TET2	W954X	87110	16	-	-	0.000162	0.002327	0.000871	0.001183	1081785.1	0.000000
18	TET2	Y1337X	87145	16	-	-	0.000014	0.001537	0.001304	0.001173	1060647.1	0.000000
18	BCORL1	R784X	1319521	8	-	-	0.003080	0.018627	0.017259	0.017138	1115442.2	0.000000
19	CBL	C381Y	34073	24	0.001034	0.001371	0.001035	0.001124	0.001829	0.001252	2974576.2	0.000000
19	TP53	H168R	43545	24	0.000705	0.001006	0.000789	0.003275	0.003164	0.002926	1060647.2	0.000000
20	KRAS	G12D	521	24	-	-	0.000142	0.000946	0.001437	0.000974	2974576.1	0.000000
20	TET2	A1381fs	-	8	0.005228	0.003205	0.003524	0.039980	0.025601	0.035660	1108362.1	0.000000

Supplementary Table 5 Summary of variant VAFs detected by droplet digital PCR in sorted hematopoietic compartments.

Participant ID	Gene	Amino Acid	COSMIC	Collection	Bulk	B	T	M
4	DNMT3A	R320X	133724	1	0.0068	0.0048	0.0005	0.0019
				2	0.0063	0.0063	0.0012	0.0039
5	DNMT3A	R635Q	1583088	1	0.0009	0.0006	0.0005	0.0005
				2	0.0020	0.0020	0.0015	0.0018
7	DNMT3A	P904L	87007	1	0.0020	0.0025	0.0001	0.0009
				2	0.0088	0.0135	0.0018	0.0037
8	TET2	G1288S	110780	1	0.0017	0.0000	0.0000	0.0006
				2	0.0016	0.0000	0.0000	0.0001
9	DNMT3A	S714C	87011	1	0.0282	0.0060	0.0018	0.0028
				2	0.0385	0.0066	0.0044	0.0058
11	DNMT3A	W860R	231568	1	0.0007	0.0000	0.0000	0.0001
				2	0.0022	0.0000	0.0000	0.0019
12	DNMT3A	G543A	256033	1	0.0007	0.0000	0.0043	0.0048
				2	0.0027	0.0015	0.0123	0.0121
13	DNMT3A	R882H	52944	1	0.0002	0.0000	0.0000	0.0001
				2	0.0020	0.0018	0.0000	0.0002
15	TP53	P278H	43755	1	0.0143	0.0234	0.0046	0.0035
				2	0.0378	0.0231	0.0358	0.0084
16	DNMT3A	I705T	1583102	1	0.0753	0.0760	0.0084	0.0350
				2	0.0545	0.0701	0.0218	0.0408
18	TET2	W954X	87110	1	0.0002	0.0000	0.0000	0.0000
				2	0.0012	0.0033	0.0004	0.0003
19	TP53	H168R	43545	1	0.0008	0.0007	0.0000	0.0000
				2	0.0029	0.0027	0.0006	0.0000
20	KRAS	G12D	521	1	0.0001	0.0000	0.0002	0.0007
				2	0.0010	0.0037	0.0001	0.0036

Supplementary Table 6 Primer sequences for library preparation.

Primer Name	Sequence
i5 16N Random	AATGATACGGCGACCACCGAGATCTACACNNNNNNNNNNNNNNNNACACTCTTTCCCTACACGACGCTCTTCCGATCT
P5	AATGATACGGCGACCACCGA
P7	CAAGCAGAAGACGGCATACGA
